# Assessing the macroeconomic and social impacts of slow steaming in shipping: a literature review on small island developing states and least developed countries

**DOI:** 10.1186/s41072-023-00131-2

**Published:** 2023-02-27

**Authors:** Seyedvahid Vakili, Fabio Ballini, Alessandro Schönborn, Anastasia Christodoulou, Dimitrios Dalaklis, Aykut I. Ölçer

**Affiliations:** World Maritime University, Malmö, Sweden

**Keywords:** Air emissions, Decarbonisation of international shipping, IMO's short term measure, Least Developed Countries, Slow steaming, Small Island Development States, Sustainable shipping, Transportation cost

## Abstract

The International Maritime Organisation (IMO) has adopted the Energy Efficiency Existing Ship Index (EEXI) and the Carbon Intensity Indicator (CII) as short term measures for decarbonisation of the shipping industry; the IMO also made the collection of relevant data and associated reporting of the indicator mandatory from January 2023. However, many existing ships do not meet the EEXI and CII “targets” and cannot invest in other technologies to meet the relevant requirements. Given the various barriers to energy efficiency, the application of slow steaming may be a measure to effectively meet EEXI and CII requirements. A qualitative systematic literature review was conducted on the potential macroeconomic and social impacts of slow steaming on states, with a special focus on Small Island Development States and Least Developed Countries, when used as the primary modality of reducing GHG emissions from shipping. This effort includes peer-reviewed studies and studies from the gray literature, many of which include examples that borrow data from the aftermath of the economic crisis that was manifested in 2008. The vast majority of those studies is focused on the economic cost-effectiveness or impact on transportation costs when using slow-steaming as a means of reducing marine fuel consumption. Moreover, a number of these studies were relying on modeling techniques, by using a limited number of ships and associated routes to determine the effects of slow-steaming. A reasonable degree of agreement emerged from the literature that a reduction in transportation costs results from a reduction in speed, being attributed primarily to reduced fuel costs, with which it is associated. Other cost-increasing factors, such as vessel operating costs, had a less dominant effect. The literature often pointed out that the cost reduction resulting from the application of slow-steaming was unevenly distributed among maritime stakeholders. Shipping companies were the main beneficiaries of significant cost savings, but these "savings" were not always passed on to shippers.

## Introduction

Maritime transportation is associated with a contribution in carriage of 80% of cargo by volume and 70% by value; it is clearly the backbone of global trade (UNCTAD 2021). Although the shipping industry plays a crucial role in trade and supply chain as well as creating jobs, and economic progress for societies, it also clearly associated with certain negative externalities. Maritime transport was responsible for 2.89% of global Greenhouse Gas (GHG) emission in 2018, and its general trend since 2013/2014 has been for increasing GHG from shipping (9.6%) and international shipping (5.6%) (Fourth IMO GHG Study 2020). On the positive side, at the 72nd session of Marine Environment Protection (MEPC), the IMO (MEPC 2018) adopted the *Initial IMO Strategy on reduction of GHG emissions from ships* (Initial Strategy) in 2018, with a vision to phase out GHG emissions from international shipping, as soon as possible in the twenty-first century. The ambition of this strategy is to reduce average carbon dioxide (CO_2_) emissions per transport work by 40% by 2030, and by 70% by 2050 compared to 2008. The total annual GHG emissions related to international shipping are aimed to be reduced by 50% by 2050, and to reach zero before the end of the century (Vakili et al. 2022a, b, c).

Current measures addressing GHG emissions are regulated under International Convention for the Prevention of Pollution from Ships (MARPOL) Annex VI and are comprised by the Energy Efficiency Design Index (EEDI), the Ship Energy Efficiency Management Plan (SEEMP), and the Fuel Oil Consumption Data Collection System (DCS), mandating annual reporting of CO_2_ emissions. The IMO has adopted a two-tier approach to implementing decarbonisation measures, focusing first on a limited set of short-term measures, before implementing more comprehensive medium- and long-term measures. At the Marine Environment Protection Committee (MEPC) 75 meeting (MEPC 2020) a combined short-term measure was agreed, comprising a technical measure (Energy Efficiency Existing Ship Index, EEXI) and an operational measure (Carbon Intensity Indicator, CII) (Vakili et al. 2021). This approved short-term measure defines a carbon intensity reduction goal, but it does not specify the means by which it will be achieved. Amongst the guiding principles of the Initial Strategy is the need for the impacts of the measures on States to be assessed, with particular attention to developing countries, especially small island development States (SIDS) and least developed countries (LDCs) (MEPC 304(72)).

The aim and the originality of this paper is to identify the potential impacts of slow steaming, when used as a short-term measure. MEPC.1/Circ.885 (MEPC 2019), provides a description of the procedure for the impact assessment of the proposed measures. The impact assessment must be simple, inclusive, transparent, flexible, evidence-based and measure-specific, as well as proportionate to the complexity and nature of the candidate measures in alignment with consideration of the candidate measures development. Additionally, the initial impact assessment must pay particular attention to the needs of developing countries, especially Small Island developing States (SIDS) and least developed countries (LDCs). Moreover, the impact assessment should consider, as appropriate, inter alia (1) geographic remoteness of and connectivity to main markets; (2) cargo value and type; (3) transport dependency; (4) transport costs; (5) food security; (6) disaster response; (7) cost-effectiveness; and (8) socio-economic progress and development.

The above criteria formed the basis for the analysis of those articles identified in the relevant literature review. As already pointed out, the goal-based measure involves a limitation of operational emissions, which means that the speed and other operational characteristics must follow the carbon intensity target in practice. Although slow steaming is not equivalent to the goal-based measure, the target-based measure can lead to speed reductions. For existing vessels, the most obvious operational measure that can be used to meet the operational goal-based objectives is a reduction in speed, and a similar situation applies to technical measures, since EEXI or power reduction would lead to a reduction in speed, and the owner, operator or charterer of the vessel is the relevant decision-maker (Vakili et al. 2022a).

The initial impact assessment in this research effort seeks to identify both positive and negative potential impacts of slow-moving traffic and to analyse the magnitude of the effects on transport costs, trade and those relating to Gross Domestic Product (GDP). In addition, the initial impact assessment seeks to evaluate whether the measure is likely to lead to disproportionately negative impacts and, if so, how they can be managed (e.g., avoided, addressed or mitigated), if appropriate.

This document is structured as follows: "Introduction" section provides the necessary Introduction; "Methodology" section describes the methodology of the study that was based on a systematic literature review; "The analysis of the peer-reviewed and grey literature" section summarises the analysis of the peer-reviewed and grey literature, and discussions and conclusions are presented in "Discussion" and "Conclusions" sections respectively.

## Methodology

This qualitative systematic literature review aimed at identifying the potential impact of short-term measures of the IMO’s Initial Strategy—more specifically slow steaming- on States, as reported in the literature. Particular attention was paid to the needs of developing countries, especially SIDS and LDCs, with regard to the eight impact criteria provided in MEPC.1/Circ.885 (MEPC 2019). This literature review process includes relevant peer-reviewed articles, and various relevant grey literature (non-peer reviewed) documents. A systematic process was used to collect peer reviewed literature, as well as grey literature, in order to provide an objective means of identifying the sources.

The literature was identified by keyword searches in the following abstract and citations databases: Scopus, Science Direct, Google Scholar, Research Gate, and EBSCO. The specific keywords and their combinations used were as follows: “slow steaming”, “speed reduction”, “impact assessment”, “engine power limit”, “shaft power limit”, “Developing Countries”, “SIDs”, “LDCs”, “connectivity”, “geographic remoteness”, “cargo value”, “cargo type”, “transport dependency”, “transport cost”, “freight”, “inventory cost”, “food security”, “disaster response”, “socio-economic progress and development”, “logistic” and “cargo quality”.

**Fig. 1:**
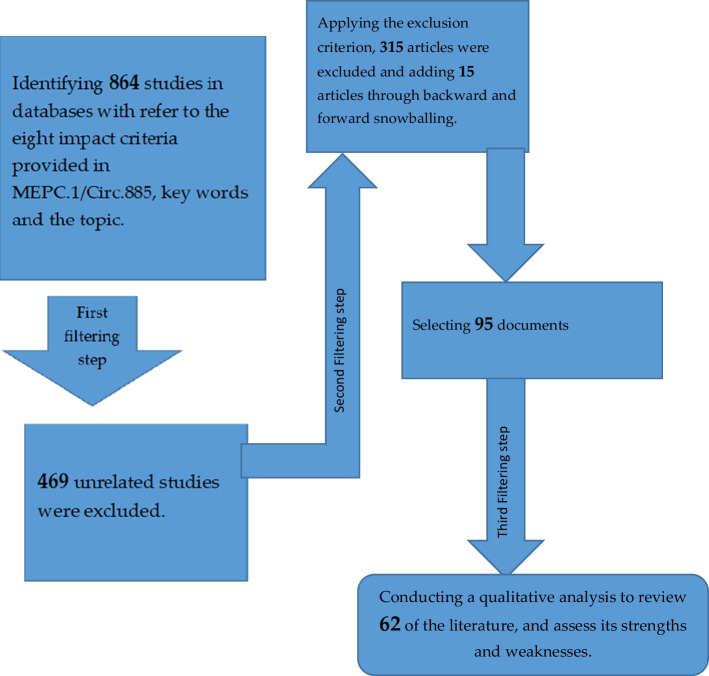
The flow chart of the methodology

A total of 864 works in eight categories were initially identified. To select the most relevant articles, two criteria for inclusion and exclusion were considered (see Table 1). In the first filtering step, where titles, conclusions, abstracts and keywords were analysed and considered, 469 studies were excluded and only 395 articles that were found to be relevant according to exclusion criterion one (Table 1). In the second filtering step, the exclusion criterion two (Table 1) was applied after reading and analysing the whole text. In this second step, 315 articles were found not to explicitly contribute to the topic, and were then excluded. In addition, 15 articles were included through backward and forward snowballing, which resulted in a total of 95 documents. In a third filtering step, any works older than 2003 were excluded, leaving 62 articles in the final selection. With the help of figure one, the various filtering steps that served the methodology are summarised (Fig. 1).

**Table 1:** Inclusion and exclusion criteria

Criterion one (Filtering step 1)	Criterion two (Filtering step 2)	Criterion three (Filtering step 3)
Peer reviewed articles and high quality conference papers, books, industrial and technical reports, which are relevant to research questions and address slow steaming within the eight impact criteria provided in MEPC.1/Circ.885	Non-English studies, duplicate articles, non-peer reviewed articles, low quality industrial and technical reports, and articles that not totally covered the topic	Exclude texts older than 2003

## The analysis of the peer-reviewed and grey literature

**Fig. 2:**
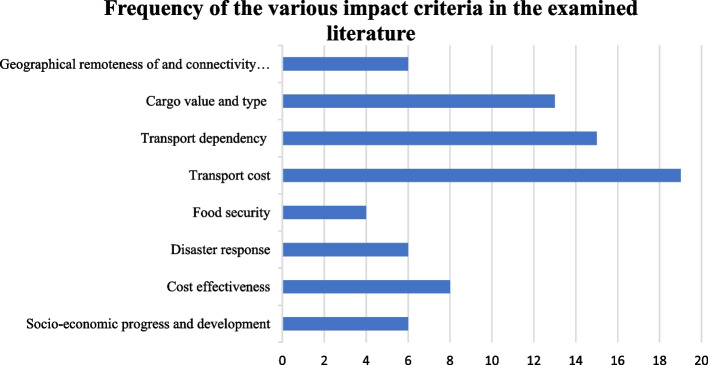
Frequency of the various impact criteria in the examined literature

This section contains the analysis of the peer-reviewed literature, along with the identified relevant grey literature. Findings are grouped with regards to each impact criterion by referring to a selection of relevant findings from the wider literature portfolio. The literature reviewed showed a stark variation in the frequency with which the literature treated the various impact criteria (Fig. 2 and Table 2). It was found that the majority of studies addressed the impact of slow-steaming on transport dependency and transport costs. A high number of studies considered cargo-value and type when discussing potential impacts. An intermediate number of studies discussed cost-effectiveness, socio-economic progress and development, and geographic remoteness and connectivity to main markets. It is also interesting to note that very few studies addressed the issues of food security and disaster response. The frequency with which the impact criteria were observed in the literature, was a mere observation of this study, and to some extent indicative of gaps in the literature. It is by no means representative of a relative importance amongst any of the impact criteria.

**Table 2:** List of the literature, considering slow steaming within eight criteria

Geographic remoteness of and connectivity to main markets	Cargo value and type	Transport dependency	Transport cost	Food security	Disaster response	Cost-effectiveness	Socio-economic progress and development
Hummel’s and Schaur (2012)	Hämäläinen (2014)	APEC (2019)	Zanne et al. (2013)	Carson (2016)	Psaraftis and Kontovas (2020)	Lindstad et al. (2012)	UNCTAD (2014)
Maloni et al. (2013)	Psaraftis and Kontovas (2014)	OECD (2014)	Psaraftis and Kontovas (2010)	Karampampa (2014)	UNCTAD (2014)	Faber et al. (2012)	Sanchez et al. (2003)
Wilmsmeier and Sanchez (2009)	Jivén et al. (2020)	Zanne et al. (2013)	Dagkinis and Nikitakos (2015)	Soyer, Tettenborn (2016)	Mimura (2013)	Finnsgård et al. (2020)	Wilmsmeier and Sanchez (2009)
Centre for International Economy (Argentina) (2020)	CEI (2019)	Wilmsmeier and Sanchez (2009)	Cepeda et al. (2017)		OECD (2014)	Zanne et al. (2013)	Newell et al. (2015)
Ferrari et al., (2015)	Karampampa (2014)	Holland et al. (2014)	Zhao et al. (2020)		Holland et al. (2014)	Cepeda et al (2017)	Jivén, et al. (2020)
APEC (2019)	Soyer, Tettenborn, (2016)	Mallidis et al. (2018)	Gurning et al. (2017)		Robinson (2020)	Mander (2017)	Doelle and Chircop (2019)
	Kontovas and Psaraftis (2011a)	UNCTAD (2014)	Wu (2020)			Centre for International Economy (Argentina) (2020)	
	Wiesmann (2010)	Yin et al. (2014)	Armstrong (2013)			Centre for International Economy (Argentina) (2021)	
	Wilmsmeier and Sanchez (2009)	Psaraftis and Zis, (2020)	Notteboom et al. (2021)			MEPC 62/INF.^7^ (2011)	
	Zanne et al. (2013)	Hummels et al. (2012)	Pradana et al. (2020)				
	Mander (2017)	Psaraftis and Kontovas (2010)	Elzarka & Morsi (2014)				
	UNCTAD (2014)	Oliveira (2014)	Carson (2016)				
		Mundaca et al. (2021)	Finnsgård et al. (2020)				
		Psaraftis and Kontovas (2020)	Cepeda & Caprace (2015)				
		Robinson (2020)	Balcombe et al. (2019)				
			Hämäläinen (2014)				
			Valentine et al (2013)				
			Centre for International Economy (Argentina) (2020)				
			Kontovas and Psaraftis (2011b)				

It is important to highlight that a comprehensive impact assessment of all measures depends on the complexity and characteristics of the measures under examination. While it may be possible to assess the impact of measures against these eight criteria, it is difficult to identify all negative impacts and disproportionate impacts on developing countries, SIDS and LDCs. As the eight criteria are interlinked and interrelated for a more accurate and comprehensive impact assessment of measures, it is important to further define the eight impact criteria, such as transport dependence and others, and consider their interlinkages.

### Geographic remoteness of and connectivity to main markets

Several peer-reviewed articles have examined the relation of slow steaming to geographically remote and distant areas from main markets. Hummels and Schaur (2012), as well as Maloni et al. (2013), discussed the problem of increased “pipeline inventory” (i.e. goods in transit between buyer and seller), including increased transport costs due to slow steaming for areas distant to main markets. Additionally, a study of Wilmsmeier and Sanchez (2009) showed that food prices, which are correlated with transport costs, are higher in more geographically remote areas, not only by geographical distance, but also by their position in global shipping networks, for example if they are situated at the start or end of shipping lines. Another important aspect discussed in this research effort is that it is necessary to include the cost of time into the transportation costs, and thus into the cost of imported or exported food. Time increases the sailing costs for crew and auxiliary power, amount of refrigeration costs, and in the case in which it affects the type of cargo that can be transported, the price and revenue of the cargo. In the case of a speed reduction or slow steaming of ships, it may be expected that this cost would increase disproportionately for countries with longer distances to markets.

Certain national studies, like the one by the Centre for International Economy (Argentina) (CIE 2020) have shown that any short-term GHG reduction measure approved by the IMO would most probably result in increased costs of export for Argentine suppliers and reduced competitiveness of Argentina's foreign trade. These increased costs will be higher for longer transport distances and disproportionately distributed amongst countries as they largely depend on their distance from the main markets with Argentinian ports being more distant to China or the EU than US, Chinese or EU ports are amongst each other.

The impact of slow steaming on maritime services and inter-port competition focusing on the major shipping services and the effects on service patterns between Asia and Europe- was highlighted in a study by Ferrari, et al. (2015). According to their findings, slow steaming can lead to the offer of differentiated shipping services, combining fast and direct services among main hubs and cheaper and slower services calling on small ports.

Meanwhile, the APEC report (2019) investigated the economic and environmental impact of slow steaming for Intra-APEC long distant economies, as well as the parameters that need to be considered when evaluating slow steaming across varied ship types, fleet, distance, and cargo from a shipper’s/trade point of view. The document highlighted that Intra-APEC long distance trade is restricted to sea, air or combination of both and analysed the nine long-distance Intra-APEC trade flows, i.e., three dry bulk trade routes and six containerized cargo trades with various values and types of cargoes. According to this report, slow steaming is more effective for ships with higher designed speeds, such as container ships and vehicle carriers. This document, through the analysis of various scenarios, clearly concluded that emission reductions will be eroded for longer distances and super slow steaming might lead to the need of additional ships.

### Cargo value and type

Cargo value and type in relation with the speed reduction measure as a modality of reducing GHG emissions from shipping has been widely discussed in literature, with varying conclusions. Speed reduction can be beneficial for shippers when bunker prices are high and market rates are low, as well as when there is fleet over-capacity due to decreasing demand (Kontovas and Psaraftis 2011a). Hämäläinen (2014) considered that speed reduction and freight shipping could economically lead to a positive effect to shippers and cargo owners in the new fuel situation from 2015 onwards. This analysis has pointed out that there are quite vast variations between export markets due to different supply chain environments. Psaraftis and Kontovas (2014) highlighted that both fuel price and freight rates, which depend on the market conditions, have a strong effect on determining the optimum sailing speed of a vessel. Meanwhile, the effects for a specific shipping company depend on various factors such as shipping segment, geographic market, modal competition and the design of the service, and can vary from minor to significant (Jiven et al. 2020).

Speed reduction may reduce short-term operating costs and this could have an impact on the final customers (Carson 2016). Additionally, this issue could have a potential significant implication for shipping customers, particularly those shipping perishable goods such as food (Karampampa 2014; Soyer and Tettenborn 2016). This implies that the increase in transport costs due to speed reduction will also vary depending on the commodity type and on whether this cost reduction can be passed-through to the producers or other actors of the supply chain. As an example, in the case study of soybean meal exports to the Netherlands, 11–41% of the cost reductions were not passed through to the other actors; however, a reduction of 5–23% of the costs were passed through to the other actors (CEI 2019). There is also a relation between slow steam measure and transportation time (Wiesmann 2010). In fact, cargo owners have to accept that the transportation time of their goods will be increased slightly and at the same time, the carriers need to adapt their trade schedule. Wilmsmeier and Sanchez (2009), also highlighted the importance of including the cost of time into the transportation costs, and thus into the cost of imported or exported food.

Although many studies show that the overall benefits of slow steaming overweigh the costs, shippers will have their individual perspectives on the practice, which may vary with type and volume of the cargo, as well as the availability of credit facilities (Zanne et al. 2013). Additionally, according to Mander (2017), slow-steaming may benefit carriers in reducing fuel costs, but this benefit may not be passed on to shippers who may suffer from increased transit times of the goods, leading to unequally distributed advantages and disadvantages. The risk of increased transit times to the quality of perishable goods is also another important factor that must be considered, especially for SIDS and LDCs in terms of global markets, such as costs and connectivity issues, as well as affecting the reliability of transport and logistics services and impact on cargo value (UNCTAD 2014).

### Transport dependency

Although the majority of studies addressed the transport dependency criterion, less attention has been paid to the impact of slow steaming on transport dependency for SIDS and LDCS. SIDS regroup a collection of countries that are diverse in many aspects, including in terms of their geographical location and respective levels of development. Despite some differences in the profile, structure and flows of their trade, SIDS share a number of common features from an international transport perspective: geographic remoteness from their main trade partners; limited volumes of trade; trade imbalances stemming from a heavy reliance on imports; and low volumes of exports highly concentrated in a few products (OECD 2014; Psaraftis and Zis 2021). As highly open economies, most SIDS are particularly dependent on their foreign trade and suffer from a strong exposure to external variations, including global or regional financial and economic crises. Also, due to their geographical location in areas of strong weather and seismic events, many SIDS find themselves amongst the most vulnerable territories in terms of exposure to natural hazards and foreseeable impacts of climate change (Robinson 2020). Both economic and environmental risks have significant bearings on SIDS’ transport systems in terms of reliability and costly operation. UNCTAD (2014), highlighted some of the related obstacles faced by transport services connecting SIDS to global markets, such as costs and connectivity issues, transport dependency, as well as disruptive weather related events affecting the reliability of transport and logistics services. Transport costs of SIDS trade are comparatively high because small volumes of trade have to travel long and indirect routes to reach distant markets.

Taking the above into consideration, Holland et al. (2014), provided context for an emerging discussion on the relationships between global CO_2_ and other pollutant emissions, in particular the contributions from global shipping, and the sea transport scenario for Pacific Island countries (PICs). The Pacific is the most dependent region on imported fuels; a major inhibitor of sustainable development for PICs. Although most operators in PICs already employ slow steaming, considering that fuel costs are the major component of shipping operators’ costs one of the options considered to reduce this cost is employing further slow steaming. This effort highlighted the problem of missing data and records in order to perform accurate impact analysis of the potential measures in order to mitigate the effect of GHG emissions in PICs. Sea transport is a critical need; the region’s transport issues are unique—small and vulnerable economies, long distances, and old ships of poor standard, entirely fossil fuel dependent and minute contribution to global emissions.

Additionally, an APEC report (2019), investigated the results of the economic and environmental impacts of slow steaming for Intra-APEC long distant economies, as well as the parameters that need to be considered when evaluating slow steaming across varied ship types (two classes of container and bulk ships), fleet, distance, and cargo from a shipper’s/trade point of view. That document highlighted that Intra-APEC long distance trade is restricted to sea, air or combination of both. The document analyses the nine long-distance Intra-APEC trade flows, i.e. three dry bulk trade routes and six containerized cargo trades with various values and types of cargoes. Depending on the commodity value and type, the study found slow steaming to have a different impact. For low valuable and non-perishable products, the study found the delay due to slow steaming to be minimal. However, for high value perishable goods or fast-moving consumer goods the study found the impact due to the delay caused by slow steaming to be considerable and that it may result in a shift to higher carbon intensive transport modes, such as air freight. Moreover, slow steaming is more effective for ships with higher designed speeds such as container ships, vehicle carriers. The document through analysis of various scenarios showed that in longer distances emission reductions will be eroded, as well as the implementation of super slow steaming might lead to requiring additional ships to ensure the service is maintained.

A certain number of the sourced articles examined the impact of slow steaming on transport dependency through analyzing the impact of slow steaming on cost. Zanne et al. (2013), highlighted that transport dependency is amalgamating with transport cost and Wilmsmeier and Sanchez (2009), conducted an analysis of the influence of transport, namely freight rates on food prices. The study carried out a regression analysis on how transport cost is affected by various factors including the maritime transport dependency and centrality of a country in the shipping liner network, allowing to investigate the impact of centrality on transport costs. This analysis showed that food prices, which are correlated with transport costs are higher in more geographically remote areas (which has more dependency to the maritime transportation), not only by geographical distance, but also by their position in global shipping networks. Other studies showed that slow steaming results to voyage cost reduction and it is not always correlated with transport distance with the largest delays occurring for the shippers on the last segment of the voyage (Mallidis et al. 2018). Additionally, a study on container ships sailing on the East Asia—North Europe trade route showed that both fuel price and a price on carbon lead to a reduction in the optimum sailing speed. The authors concluded that both an increase in fuel price or an increase in the price for CO_2_ emissions should cause ship owners to reduce their sailing speed, and that this was correlated with a positive environmental impact in terms of GHG emissions reduction (Yin et al. 2014).

Hummels et al. (2012), estimated that each day in transit is equivalent to an ad-valorem tariff of 0.6 to 2.3 percent and that the most time-sensitive trade flows are those involving parts and components trade. These results suggest a link between sharp declines in the price of air shipping and rapid growth in trade as well as growth in world-wide fragmentation of production. Psaraftis and Kontovas (2010), analysed the cost of shipping under varying conditions of charter rates and ship speed. The study built a model to consider the modal split between shipping and one land-based transportation route. It was found that according to the model used, if the speed of ships was reduced at constant freight rates, then more cargo would be shifted to the land-route. Meanwhile, number of papers discussed the transport dependency through the possibility of modal shift in case of slow steaming and its negative externalities. As an example, slow steaming may encourage some cargoes to shift to other, faster modes of transport, including road, rail or air (Psaraftis and Zis 2021), which can lead to more air emissions to the environment as the case of modal shift to air freight is 47 times more carbon intensive than a container ship (APEC 2019).

Other studies considered the determinates of freights on the main shipping route. As an example, Oliveira (2014), analyzed the determinants of freight rates on main shipping routes linking Europe to the rest of the world. According to the study freight rates across routes can vary due to specific characteristics of the routes in terms of fuel cost, amount of transshipment, trade volume, connectivity and port infrastructure. Major routes have an advantage in terms of fuel cost, not only due to maritime distance, but also because of the deployment of the most efficient vessels on these routes. The amount of transshipment also affects the freight rates that tend to be higher for routes with low trade volume. In addition, setting rules for carbon reduction in shipping is another factor that is considered to be an effective measure in setting freight rates. Mundaca et al. (2021), highlighted the potential impacts of carbon taxation of international transport fuels on CO_2_ emissions and trade activity, focusing on maritime transport. According to the findings, the bunker-price elasticities of products range from -0.003 to -0.42 and the largest impacts of carbon taxes on maritime trade are on the products with the lowest sales values in relation to their weight. A global tax of $40 per ton CO_2_ tax would reduce carbon emissions by about 7% for the heaviest traded products transported by sea with the greatest CO_2_ emission reductions being for products with particularly low value-to-weight ratios such as fossil fuels and ores. Their conclusions suggest that an increase in the bunker fuel price would lead to substantial reductions per ton-km for internationally traded products reducing at the same time the bunker fuel consumption and carbon emissions from international shipping.

### Transport cost

The majority of the peer-reviewed papers examined the impact of slow steaming on transport costs, with many of them analysing the direct impact on vessels’ operational costs and others following a more holistic approach and discussing how slow steaming would affect the whole supply chain and the various actors involved. The important benefits of applying slow steaming in reducing fuel consumption and, therefore, fuel costs and harmful emissions were highlighted in the findings of Zanne et al. (2013). Psaraftis and Kontovas (2010) and Dagkinis and Nikitakos (2015), also analysed the cost of shipping under varying conditions of charter rates and ship speed and concluded that slower speed was the preferred option in terms of costs if the charter rates were low, whereas higher speeds were preferred at higher charter rates. Cepeda et al. (2017), also conducted sensitivity analyses with respect to bunker prices and the costs of new sheets to supplement the fleet, which showed that cost reductions favoured the use of slow steaming if bunker prices are high, and favoured operation at original speed (assuming constant transport work) if the capital expense of new vessels was high.

Zhao et al. (2020), carried out an analysis of the loss aversion mechanism employed by decision makers at shipping companies in order to determine the optimal sailing speed and recommended that shipping companies should reduce sailing speed under conditions of high fuel prices, low compensation costs required to be paid to customers for late deliveries, and when there were few cargoes onboard the vessels. Gurning et al. (2017), also investigated which ship speed is most optimal for shipping companies by considering technical and operational, financial and also environmental aspects and supported that the ship's speed is the most important factor in ship's operational cost and revenue, and operating in optimum speed could help the ship-owners save costs. The analysis recommended super slow steaming as the optimal speed for container vessels. Extra slow steaming and slow steaming alternatives are placed in second and third ranks, respectively and full speed is placed as the last optimal speed for container vessels. According to Wu (2020), carriers will reduce ship speed if the saved fuel cost outweighs the incurred capital and operating costs, and slow steaming can save fuel consumption for carriers even if additional ships may be required to maintain sailing frequency. Moreover, it was highlighted that the optimal speed will be increased by the ship size, and if the carriers intend to deploy slow steaming the optimal strategy is to call extra ports with extra ships.

Armstrong (2013), found slow-steaming to be the most effective measure for the reduction of fuel consumption and suggested that, even though the implementation of slow-steaming was the result of commercial optimisation in the light of the economic recession of 2008–2009, it would be prudent to continue the practice of slow-steaming even when the world economy recovers, in order to maintain the significant fuel efficiency benefits in the interest of reducing GHG emissions. Notteboom et al. (2021), investigated and compared the temporal and spatial sequences of the supply and demand shocks of COVID-19 and the 2008–2009 financial crisis on container ports and the container shipping industry. According to their findings, adaptation mechanisms to these shocks—such as slow steaming—have not been applied at the same extent during the financial crisis and COVID-19. More specifically, slow steaming that was extensively used as a cost-saving strategy during the financial crisis of 2008–2009 was not a part of the capacity management efforts during COVID-19, as this strategy was already in place and there was little room left for further speed reductions.

Pradana et al. (2020), used analytical relations to calculate the shipping costs for cargo ships operating in Indonesia (those that connect the more remote parts of eastern Indonesia with western Indonesia). Assumptions for ship speed were made and divided into three scenarios: In the first scenario the shipping costs were calculated using real voyage data made available by the Ministry of Transportation of the Republic of Indonesia. In the second scenario, the ship speed was assumed to be reduced based on reports about the weather conditions (wind and waves) using an assumption about ship speed loss as a function of weather. In the third scenario the ship speed was reduced further, not only as a result of the weather conditions, but also as a result of a further reduction of speed of either 10% or 12%. The results showed that the lowest transport costs were calculated for the third scenario, which simultaneously implemented a reduction of ship speed due to weather and slow steaming. For two out of the three shipping routes used in this investigation, the lowest transport cost was achieved for the 10% speed reduction in the third scenario.

A number of studies analysed the impact of slow steaming on the different actors of the supply. According to Elzarka and Morsi (2014) the impact of slow steaming on cost is undeniable, but supply chain lead times become longer and alternative supply sourcing options need to be examined (e.g. near-shoring or on-shoring). Carson (2016), stated that, even though speed reduction has reduced short-term operating costs for ship operators, shippers have not benefited from slow steaming to the same extent as carriers have not passed on savings from reduced shipping costs via lower freight rates. Similar findings come from the study of Finnsgård et al. (2020); the authors conducted a case study on six Swedish companies in various industries using shipping to transport their goods and focused on the impact of slow-steaming on the shipper, and not on the ship owner. The study noted that in principle there is a negative abatement cost to GHG abatement from slow-steaming. According to their findings, all companies reported that they felt increased transit times associated with slow-steaming, with only one of the companies receiving a recognisable reduction in transport cost, as a result of the slow-steaming practices. This means that all the cost-reduction due to this practice remained with the carrier.

Cepeda and Caprace (2015), assessed in particular whether the reduced transportation work due to slow-steaming or ultra-slow-steaming would still be profitable although new ships would need to be purchased to restore the transportation work to its original value. It was found that both slow-steaming and ultra-slow steaming were more profitable than the current practice, and that costs for purchasing new vessels were more than offset. Zanne et al. (2013), elaborated on the GHG and transport cost reduction benefits of slow steaming in the wake of the economic recession of 2008–2009 and concluded that slow-steaming is the most cost-effective measure of reducing fuel consumption and air emissions, but shippers will have their individual perspectives on the practice, which may vary with type and volume of the cargo, as well as the availability of credit facilities. Balcombe et al. (2019) found out that the average GHG savings due to slow steaming are around 19%, if the addition of further vessels to make up for the lost transport work is considered, and underlined that, while slow steaming results in considerable fuel savings, the cost reduction is not always felt by the cargo owner and slow-steaming practices may require some form of regulation. Hämäläinen (2014), examined how slow steaming in freight shipping can bring economically positive effects to shippers and cargo owners in the new fuel situation from 2015 onwards. According to this analysis, slow steaming has been used to tackle the negative economic impacts of the Sulphur Directive in the short sea shipping routes, but positive impacts of slow steaming vary from market to market. Valentine et al. (2013), also suggested that slow steaming consists of one of the measures adopted by the shipping industry as a response to rising fuel costs.

The 2020 report of the Centre for International Economy (Argentina) (2020) provides an analysis of the economic impact of energy efficiency measures in shipping on the cost of Argentine exports. According to this analysis, speed reductions of 10–30% could reduce actual cost of transportation, even if additional ships would be needed to maintain a constant transportation rate. The study highlights the importance of this costs reduction to be passed-through to the producers; for example, for the case of soybean exports to China, producers would face a cost increase by 11–37% or an actual reduction in their costs by 6–26% depending on whether the transport costs reductions would be passed through.

Kontovas and Psaraftis (2011), investigated the scenario of reducing emissions along the maritime intermodal container chain and explored the potential effects of speed reduction of container vessels on reducing emissions and fuel consumption. They investigated possible ways to decrease time in port since slow steaming increases the time of vessels at sea focusing on the potential to reduce berthing and waiting times at container ports to keep the total turnaround time constant. According to their findings, speed reduction can be beneficial for shippers when bunker prices are high and market rates are low and when there is fleet over-capacity due to decreasing demand. Concerning the cost-effectiveness of speed reduction, the authors pointed out that it is an overall more profitable option for the operators depending on the additional costs of deploying the extra vessels. For the charterers, speed reduction relates to increased in-transit inventory costs that are proportional to the value of the cargo.

### Food security

A number of studies have identified slow-steaming as having a potential negative impact on food prices and food security. Some articles argued that slow steaming may have significant implications for the shipping customers of perishable goods such as food (Karampampa 2014; Carson 2016; Soyer and Tettenborn 2016) and Wilmsmeier and Sanchez (2009), conducted an analysis of the influence of transport, namely freight rates on food prices. In the introduction freight rates of the first part of this millennium leading up to the start of the economic crisis in the last quarter of 2008 are compared with food prices, indicating considerable correlation. The study notes that since the food importer bears the price of the transport costs, increased transport costs would be expected to have a considerable economic and societal impact on the costs and consumption of food. People with low incomes would be particularly vulnerable to an increase in food costs deriving from transport cost, and may thus affect food security. This analysis shows that food prices, which are correlated with transport costs are higher in more geographically remote areas, not only by geographical distance, but also by their position in global shipping networks, for example if they are situated at the start or end of shipping lines. Another important aspect discussed in this article is that it is necessary to include the cost of time into the transportation costs, and thus unto the cost of imported or exported food. Time increases the sailing costs for crew and auxiliary power, amount of refrigeration costs, and in the case in which it affects the type of cargo that can be transported, the price and revenue of the cargo. In the case of a speed reduction or slow steaming of ships, it may be expected that this cost would increase disproportionately for countries with longer distances to markets.

### Disaster response

SIDS are heavily dependent on marine transportation and face common economic and development challenges. Geographical remoteness, the higher shipping costs associated with the low connectivity to main markets, the need for transhipment, and the transport dependency particularly during emergencies are some of the main concerns of SIDS (Psaraftis and Zis 2021).

Most SIDS are vulnerable to natural hazards (UNCTAD 2014), and like the other developing countries climate change and population growth is increasing the vulnerability of SIDS to disaster risk (Mimura 2013). On one hand, the larger exposure of SIDS to the impacts of climate change and natural disaster and on the other hand lack of ability of SIDS to respond, make SIDS among the most vulnerable countries in the world (OECD 2014). Connectivity of SIDS with the global community and international trade is crucial, to fulfil the essential needs of SIDS. Shipping is the "lifeline" and plays an important role for SIDS in essential connectivity and disaster response. Although the topic is crucial for SIDS, there is a lack of study, and neither papers nor case studies provide any analytical information on the extent of the importance of maritime transportation and the negative impacts of the implementation of slow steaming in relation to response in disasters.

As open and small economies, SIDS are also vulnerable to global economic and financial shocks. OECD (2014), highlighted that short-term disaster response must be linked to long-term financial support for resilience building and financing, and Holland et al. (2014), provided context for an emerging discussion on the relationships between global CO_2_ and other pollutant emissions in particular the contributions from global shipping, and the sea transport scenario for PICs. The Pacific is the region most dependent on imported fuels, which is a major inhibitor of sustainable development for PICs. The paper highlighted that sea transport is a critical need and the region’s transport issues are unique, small and vulnerable economies, long distances, and old ships of a poor standard, entirely fossil-fuel dependent and minute contribution to global emissions.

Robinson (2020), highlighted that although SIDS are among the least responsible of all nations for climate change, they are likely to suffer strongly from its adverse effects and could in some cases even become uninhabitable. Ninety per cent of the SIDS are in the tropics, and almost all SIDS depend heavily on fossil fuels. The use of fossil fuels includes not only power production and the desalination of water, but also transport, including tourists as well as the transfer of goods.

### Cost-effectiveness

A rather intermediate number of studies discussed the cost-effectiveness of vessel speed reduction as a means of reducing GHG emissions from shipping with varying conclusions. These studies found that speed reduction was a cost-effective means of reducing GHG emissions, in that it comes at no economic cost, or even at a negative GHG abatement cost. Part of this group of studies was document MEPC 62/INF.7 (2011) which concluded that a 10% or 20% speed reduction would both have negative abatement costs, but that a 10% speed reduction was most cost-effective. Lindstad et al. (2012), concluded at a reduction of GHG emissions of up to 28% being possible at zero abatement costs. The same study also concluded that global GHG emissions could be reduced by 30% over this time span at zero abatement cost, by increasing the size of the vessels. This suggests that while slow-steaming was estimated to have a negative abatement cost, the abatement potential of using larger vessels was even larger. Additionally, Finnsgård et al. (2020) highlighted that in principle the abatement cost of slow-steaming is negative and if the average speed of vessels in 2007 would be reduced to 85%, benefits would outweigh the costs by 178–617 billion USD in the period up to 2050, depending on fuel prices (Faber et al. 2012).

However, a number of studies showed a more balanced picture. Zanne et al. (2013) highlighted that while studies showed that the overall benefits of slow steaming overweigh the costs, shippers will have their individual perspectives on the practice, which may vary with type and volume of the cargo, as well as the availability of credit facilities. The authors further mentioned the potential for energy efficiency in propulsion and auxiliary power to contribute to a reduction in fuel consumption, and also highlighted the possibilities of using renewable energy such as wind and solar power, battery electric hybrid propulsion systems and alternative fuels to replace part of the energy currently derived from fuels. The authors concluded that slow-steaming is not the only way of reducing fuel consumption and air emissions, but that it appeared to be the most cost-effective measure.

Cepeda et al. (2017) showed that slow steaming and ultra-slow steaming reduced CO_2_ emissions per transport work by 51% and 85% respectively, whilst reducing at the same time SOx emissions by 43% and 78% respectively and operating costs of the fleet by 1% and 34% respectively. The fleet was supplemented with new ships in order to maintain the transport work at more or less a constant level (± 7%). The study also conducted sensitivity analyses with respect to bunker prices and the costs of new ships to supplement the fleet, which showed that cost reductions favoured the use of slow steaming if bunker prices were high, and favoured operation at original speed (assuming constant transport work) if the capital expense of new vessels was high.

A number of studies found that slow-steaming could have severe economic costs. Mander (2017), highlighted the fact that slow-steaming may have a wider impact on supply-chains beyond shipping, with businesses operating lean 'just-in time' supply chains most affected. Slow-steaming may benefit carriers in reducing fuel costs, but this benefit may not be passed on to shippers who may suffer from increased transit times of the goods, leading to unequally distributed advantages and disadvantages. The risk of increased transit times to the quality of perishable goods is also mentioned. Slow-steaming requires coordination of the larger logistics network in order to ensure sufficient warehouse space and coordination with other modes of transport. The author also mentioned the impact of slow-steaming on ship crews, increasing their transit times and potentially requiring potential changes in duties onboard.

The 2020 report by the Centre for International Economy (Argentina) (2020), provided an analysis of the economic impact of energy efficiency measures in shipping on the cost of Argentine exports. Three energy efficiency measures were considered: 1. operational measures such as speed optimisation and speed reduction, as well as improvement of port operations, 2. technical measures such as energy efficiency measures and the use of low-carbon alternative fuels, and 3. the use of market-based measures such as negotiable emissions permits and emission taxes. Two case-studies using soybean product exports to China and the Netherlands were used to evaluate the costs of the energy efficiency measures. The study observed that having to pay for CO_2_ emissions would result in a 10–36% increase in transport costs for the case of Argentine soybean exports. The study also observed that speed reductions of 10–30% could reduce actual cost of transportation, even if it was considered that additional ships would be needed to maintain a constant transportation rate. Crucially, the study noted that it depends on whether this cost reduction can be passed-through to the producers, on whether it would increase their cost by between 11 and 37% or in fact reduce their costs by 6–26% for the case of soybean exports to China. In the case study of soybean meal exports to the Netherlands, similar values were calculated: An increase of 11–41% in case the costs reductions were not passed through and a reduction of 5–23% if the costs were passed through. Finally, it was noted that there exists a risk that increases in transport costs could result in agricultural produce suppliers from other countries may step in and supply the markets instead, but that this depends on how much of the cost-savings due to fuel reduction are passed-on to the charterers.

Furthermore, the 2021 paper by the Centre for International Economy (Argentina) (2021), argues that GHG reduction measures approved by the IMO would result in increased costs of export for Argentine suppliers with high probability and this would result in a reduced competitiveness of Argentina's foreign trade. It was also argued that these costs would be higher for longer transport distances, and that therefore increases in costs would be disproportionately distributed amongst countries, and depend on their distance from the main markets. The paper aims to provide preliminary statistical data for analysing the impact of the short-term GHG reduction measure on trade. First, data was provided on the fraction of trade executed by shipping, which in the case of Argentina is much higher at 75% than the United States (46%) or the EU (35%). It was also argued that Argentine ports have longer distances to China or the EU, than equivalent US, Chinese or EU ports have amongst each other. Data was also provided on the industrial sectors on which Argentina depends for exports. Argentina's exports rely predominantly on agriculture food production, fish and minerals (which are predominantly transported by ship), with the share of industrial products being relatively low at 22% of value. Finally, data was provided on the chilled and frozen meat exports, on which Argentina strongly relies. It was shown that chilled meat exports provide higher revenues, because of the higher market price for chilled meats. If shipping speeds are reduced, Argentina will be impacted by losing markets for chilled meat, and thus loose revenue. It may be able to make up trade losses to some extent by exporting frozen meat instead, if possible, but export losses would persist due to the lower market prices for frozen meat. These negative economic impacts may need to be considered in the cost-effectiveness of the measures.

### Socio-economic progress and development

Socioeconomic progress and development can be defined as the identification and inclusion of factors determining the rate of the socioeconomic progress of a country under definite conditions of its development process (Gilani and Hoseinzadeh 2021; Khodayar Sahebi et al. 2021; Hoseinzadeh and Garcia 2022; Vakili et al. 2022b). The change to a long-term slow steaming scenario need, however, a number of considerations. Most SIDS are particularly dependent on their foreign trade and suffer from a strong exposure to external variations, including global or regional financial and economic crises. Both economic and environmental risks have significant bearings on their transport systems in terms of reliability, costly operation and connectivity issues (UCTAD 2014). According to Sanchez et al. (2003), on average, 8.6% of the value of merchandise imported by the countries of Latin America and the Caribbean is spent on freight and insurance costs relating to their international carriage; this figure is 40% higher than the world average. Major differences persist within the region, with the Caribbean economies recording the highest indices.

Wilmsmeier and Sanchez (2009), highlighted in their analysis that food prices, which are correlated with transport costs, are higher in more geographically remote areas, not only by geographical distance, but also by their position in global shipping networks, for example if they are situated at the start or end of shipping lines. The effects for a specific shipping company depend on various factors, such as shipping segment, geographic market, modal competition and the design of the service, and can be varied from minor to significant (Jiven et al. 2020). In addition, according to Doelle and Chircop (2019) there is a necessity of further assessment for the overall effect of speed management, especially regarding safe navigation and economic development for SIDs and LDCs. As an example, Pacific islands due to their specific characteristics, such as severe dependency on fossil fuel, long routes, minute economies, imbalance in import and export, financial barriers, and high infrastructure cost, face greater challenges in achieving long-term sustainable shipping (Newell et al. 2015). Table 3 summarises our findings on the impact of slow steaming on countries, and most specifically, the LDCs and the SIDS.

**Table 3:** Impact of slow steaming on countries based on the eight criteria

Eight criteria	Impact of slow steaming on LDCs and SIDS
Geographic remoteness of and connectivity to main markets	Increased 'pipeline inventory' and overall transport costs for areas distant to main markets due to their geographical distance, but also their position in global shipping networks Increased export costs and reduced competitiveness of foreign trade for longer transport distances and disproportionate impact distribution for countries distant to the main markets Impact on inter-port competition focusing on the major shipping services and leading to the offer of differentiated shipping services, combining fast and direct services among main hubs and cheaper and slower services calling on small ports
Cargo value and type	The impact depends on various factors such as shipping segment, geographic market, modal competition and the design of the service, and can vary from minor to significant Potential significant implication for shipping customers, particularly those shipping perishable goods such as food Will also vary depending on the commodity type and on whether this cost reduction can be passed-through to the producers or other actors of the supply chain The risk of increased transit times to the quality of perishable goods, especially for SIDS and LDCs in terms of connectivity issues, as well as the reliability of transport and logistics services and impact on cargo value
Transport dependency	The SIDS region’s transport issues are unique—small and vulnerable economies, long distances, and old ships of poor standard, entirely fossil fuel dependent and minute contribution to global emissions If the speed of ships was reduced at constant freight rates, then more cargo would be shifted to the land-route for short distance shipping routes Slow steaming may encourage some cargoes to shift to other, faster modes of transport, including road, rail or air, which can lead to more air emissions to the environment Freight rates across routes can vary due to specific characteristics of the routes in terms of fuel cost, amount of transshipment, trade volume, connectivity, and port infrastructure. Major routes have an advantage in terms of fuel cost, not only due to maritime distance, but also because of the deployment of the most efficient vessels on these routes. The amount of transshipment also affects the freight rates that tend to be higher for routes with low trade volume
Transport cost	Important benefits in reducing fuel consumption and, therefore, fuel costs and harmful emissions Shipping companies should reduce sailing speed under conditions of high fuel prices, low compensation costs required to be paid to customers for late deliveries, and when there were few cargoes onboard the vessels The impact of slow steaming on cost is undeniable, but supply chain lead times become longer and alternative supply sourcing options need to be examined (e.g. near-shoring or on-shoring) Even though speed reduction has reduced short-term operating costs for ship operators, shippers have not benefited from slow steaming to the same extent as carriers have not passed on savings from reduced shipping costs via lower freight rates Slow-steaming is the most cost-effective measure of reducing fuel consumption and air emissions, but shippers will have their individual perspectives on the practice, which may vary with type and volume of the cargo, as well as the availability of credit facilities An overall more profitable option for the operators depending on the additional costs of deploying the extra vessels. For the charterers, speed reduction relates to increased in-transit inventory costs that are proportional to the value of the cargo
Food security	Slow-steaming having a potential negative impact on food prices and food security May have significant implications for the shipping customers of perishable goods such as food People with low incomes would be particularly vulnerable to an increase in food costs deriving from transport cost and may thus affect food security Necessary to include the cost of time into the transportation costs, and thus unto the cost of imported or exported food. Time increases the sailing costs for crew and auxiliary power, amount of refrigeration costs, and in the case in which it affects the type of cargo that can be transported, the price and revenue of the cargo
Disaster response	SIDS are heavily dependent on marine transportation and consist small and vulnerable economies that face common economic and development challenges due to geographical remoteness, the higher shipping costs associated with the low connectivity to main markets, the need for transhipment, and the transport dependency particularly during emergencies
Socio-economic progress and development	Most SIDS are particularly dependent on their foreign trade and suffer from a strong exposure to external variations, including global or regional financial and economic crises. Both economic and environmental risks have significant bearings on their transport systems in terms of reliability, costly operation and connectivity issues Pacific islands due to their specific characteristics, such as severe dependency on fossil fuel, long routes, minute economies, imbalance in import and export, financial barriers, and high infrastructure cost, face greater challenges in achieving long-term sustainable shipping

## Discussion

From a global perspective, the impact of slow steaming as an emission reduction strategy will vary from market to market and geographic regions (Valentine et al. 2013; Hämäläinen 2014). As highlighted in some studies, when implementing slow steaming in shipping, it is crucial to maintain a balance between reducing emissions and extending transit times, while taking into account customer tolerance for longer transit times (APEC 2019). If the balance is disturbed and customer tolerance is exceeded, modal shifts to modes with significantly higher carbon intensity, such as air cargo, or, in the worst case, the potential loss of trade for highly time-sensitive cargoes may occur. In line with this, the current sequences of the supply and demand shocks due to the COVID-19, which have different origins and impacts compared to the 2008–2009 financial crisis, should have been taken into consideration for further projection analysis. Slow steaming, adopted during the financial crisis of 2008–2009 in an attempt to absorb excess capacity, could not be used as a tool during the pandemic as the practice of slow steaming was largely in place as the response of shipping lines to reduced shipping volumes caused by the global financial crisis of 2008–2009 (Armstrong 2013). Additionally, slow steaming is not a measure that can be applied universally and is contextual to the technology the industry is operating with and market conditions with the newbuilding and retrofitted vessels already operating at lower speeds.

Developing, SIDS, and LDCs vary in many ways, including in terms of their geographic location, their respective level of development, and their exposure to natural disasters and the likely impacts of climate change (UNCTAD 2014), but they are all strongly affected by the negative impacts of climate change. The Pacific region is the most dependent on imported fuels and since fuel costs are the major component of ship operators' costs, they are a major constraint on the sustainable development of PICs (APEC 2019). Therefore, slow steaming is one of the options to reduce these costs and currently most operators in the region are already using it whenever possible.

In general, freight rates for SIDS and LDCs are high (De Oliveira 2014), which is due more to the monopoly control of the market by shippers than to connectivity with distance to major routes. Therefore, due to the lack of adequate policies and funding, some literature recommends considering direct and indirect compensation mechanisms to address the concerns of SIDS and LDCs. Almost all SIDS rely heavily on fossil fuels for transportation (APEC 2019) and due to barriers such as cost and connectivity issues, transportation services to connect SIDS and LDCs to global markets may be affected. Moreover, the reliability of transport and logistics services for SIDS and LDCs is unique and should be taken into account when assessing the impacts of proposed measures.

Taking into account the above and changes in the strategic behavior of market participants, further adjustment mechanisms, such as slow steaming, have been shown to lead to different outcomes and uncertainty, especially for SIDS, LDCs and developing countries. However, it would be useful to use historical data on the application of slow steaming, especially after the 2008 financial crisis, and consider a holistic, systematic, and transdisciplinary approach to assess the direct and indirect impacts of slow steaming on all maritime cluster stakeholders (Vakili et al. 2022a), especially for SIDS and LDCs.

As indicated in several studies, it is important that all actors in the supply chain adopt slow steaming (APEC 2019; CIE 2020; CIE 2021). If or when slow steaming is made mandatory, the windows of berths in ports, for example, will have to be reviewed and adapted to the new operational regime; i.e., no port fees should be charged for slow steaming. Slow steaming may also be economically problematic and practically difficult for small passenger ships and mixed passenger/cargo ships, which are a lifeline for connecting many SIDS, LDCs and microstates. Passenger ships are the only transportation option for the connections of many SIDS and LDCs. However, the impact of slow steaming on passenger ships, feeder and sub feeder transport was not adequately analyzed in the peer-reviewed papers, and the results of the impact assessments were mainly based on qualitative literature reviews, with no segmentation provided. In this context, a temporary exemption regime from slow steaming could be proposed for certain vessel types and sizes serving SIDS and LDCs, but it should be emphasized that further research is needed since SIDS are not a homogeneous group and their specific characteristics should be considered.

The research effort found that most studies focused primarily on the economic impact of the use of speed reduction by ship owners, without a proper quantitative impact assessment of the other relevant supply chain actors. As several authors suggested during the peer review, slow steaming is an important measure that improves economic efficiency and environmental sustainability, as it directly affects the amount of fuel used, which is directly proportional to fuel costs and emissions (Zanne et al. 2013). However, the economic benefit gained by the ship owner from slow steaming is not equally distributed among the different actors of the global supply chain (Mander 2017). This suggests that while slow steaming will reduce operational costs, it will simultaneously lead to longer lead times in the supply chain and that alternative supply options should be considered to overcome this challenge. For many SIDS, LDCs and even developing countries, the demand for maritime transport is inelastic. In this sense, it is questionable whether any cost savings will ultimately be passed on to shippers and states and to what extent.

Another assumption in most impact assessment studies is that overall transportation costs will decrease as a result of slow shipping and lower operating costs due to less fuel consumption (CEI 2019). However, speed reduction equates to less transportation work that may lead to the use of more or larger vessels (Lindstad et al. 2012). The impact of such a development on more air emissions, ports and overall logistics costs needs to be assessed, as a larger number of ships may lead to long waiting times at ports and higher inventory costs borne by shippers and states and inventory costs directly related to cargo value. This will make more sense for SIDS and LDCs that are geographically remote and lack good connectivity to key markets. In these states, logistics costs represent a significant portion of total transportation costs, with much transshipment required for goods to reach their final destination.

Another important factor in the impact assessment of slow steaming is cargo type. Cargo may be time-sensitive due to the nature of its industries, such as perishable goods, high-value goods, or high turnover consumer goods (FMCG), is strongly affected by changes in transportation time and will seek the option that meets the cargo's required delivery dates, which may lead to shifts to more carbon-intensive modes, such as air freight (Psaraftis and Kontovas 2010; APEC 2019; Psaraftis and Zis 2021). As highlighted in several studies, product characteristics and total transportation costs are important factors in the choice of transport mode; perishable, high-value products may prefer air transport over sea transport because the value of the product can absorb the typically higher air freight costs, and time-sensitive products, such as FMCG, are also more likely to choose air transport over sea transport to meet delivery deadlines in their supply chain. Perishable goods in particular can be very sensitive to speed reductions. Some authors suggested that the effect of reduced operating costs due to speed reductions should be examined in relation to the importance of transit time in trade goods. It is important to emphasize that transit time may be of secondary importance for bulk and simple industrial goods, but is critical for perishable goods (APEC 2019).

## Conclusions

The IMO has adopted the EEXI and CII as short term measures for decarbonisation of the shipping industry and the relevant data gathering and reporting scheme is obligatory since January 2023. However, many existing ships do not comply with EEXI and, due to their age, cannot invest in other technologies to reduce air emissions. Given the current barrier, the application of slow steaming may be an propionate measure to meet EEXI and CII requirements.

This qualitative systematic literature review was carried out as part of the comprehensive impact assessment of the amendments to MARPOL Annex VI on the approved short-term measure to reduce the carbon intensity of international shipping as approved by MEPC 75. The aim of this literature review is to identify potential impacts of the approved short-term measure on States, including developing countries, in particular, LDCs and SIDS. This effort was focused on documents related to the technical and operational implementation of the approved short-term measure, and includes relevant literature identified therein, peer-reviewed articles in the open-literature, relevant grey literature documents (non-peer reviewed documents), and any documents identified by the Steering Committee and the IMO Secretariat. The approved short-term measure defined a goal of carbon intensity reduction, rather than prescribing a means of achieving it. This literature review focussed mainly on speed reduction, on which there is ample material available, and which may be implemented with relative simplicity and negligible initial cost.

The analysis of the literature was carried out in the context of the short-term GHG reduction measure; it revealed a stark variation in the frequency with which the open literature treated the eight impact criteria. The majority of studies addressed the impact of slow-steaming on transport dependency and transport costs of speed reduction. A high number of studies considered cargo value and type when discussing potential impacts. An intermediate number of studies discussed cost-effectiveness, socio-economic progress and development, and geographic remoteness and connectivity to main markets. Only a few studies addressed the issues of food security and disaster response.

Slow steaming, as a means of complying with carbon intensity reductions in the short-term, was also found by some peer-reviewed literature studies to represent a key measure that reduces greenhouse gas emissions at high economic efficiency since it directly affects the quantity of fuel used that is directly proportional to fuel costs and emissions. On the other hand, it was a salient feature of the literature related to speed reduction, that economic benefits gained by the ship-owner were not necessarily equally distributed among the different actors of the global supply chain. It was also suggested in the literature that although slow steaming may be expected to reduce operational costs, it will at the same time result in longer supply chain lead times resulting in the need to consider alternative supply sourcing options to overcome this challenge.

The literature showed that there are certain features of transport services connecting SIDS and LDCs to global markets that are unique such as costs and connectivity issues, transport dependency as well as disruptive events affecting the reliability of transport and logistics services and need to be taken into consideration when assessing the impacts of proposed measures. Lack of a proper and up-to-date set of data and pertinent studies specifically focused on SIDS and LDCs, still constitutes a major shortcoming in order to perform a detailed and accurate impact assessment. Therefore, due to lack of data availability, most of the studies focussed on qualitative rather quantitative analysis.

Although slow steaming is expected to bring large economic and environmental benefits to the states whose economy depends heavily on international seaborne transport, an holistic and transdisciplinary approach needs to be adopted for conducting a comprehensive impact assessment. In this direction, further research on this topic should ideally consider employing methodologies and approaches to deal with conflicting objectives under trade off environment, such as from a simple Cost/Benefit Analysis to more advanced Multiple Criteria Decision Making tools.

## Data Availability

Not applicable.

## Abbreviations

CII: Carbon intensity indicator
DCS: Data collection system
EEDI: Energy efficiency design index
EEXI: Energy efficiency existing ship index
GDP: Gross domestic product
GHG: Greenhouse gas
IMO: International maritime organization
LDCs: Least developed countries
MEPC: Marine environment protection
PICs: Pacific island countries
SEEMP: Ship energy efficiency management plan
SIDS: Small island development states

## References

[CR1] APEC (2019) Analysis of the impacts of slow steaming for distant economies, APEC Transportation Group, APEC Project: TPT 03 2018A, produced by Starcrest Consulting Group for the Asia-Pacific Economic Cooperation Secretariat, December.

[CR2] Armstrong VN (2013). Vessel optimisation for low carbon shipping. Ocean Eng.

[CR3] Balcombe P, Brierley J, Lewis C, Skatvedt L, Speirs J, Hawkes A, Staffell I (2019). How to decarbonise international shipping: options for fuels, technologies and policies. Energy Convers Manag.

[CR4] Carson JK (2016) The implications of slow steaming for shipping customers

[CR5] Centro de Economia Internacional (CEI) (2019) Greenhouse gas reduction measures in maritime transport and their impact on the cost of Argentine exports.. Greenhouse gas reduction measures in maritime transport and their impact on the cost of Argentine exports, https://cancilleria.gob.ar/es/cei/publicaciones

[CR6] Centre for International Economy (2020) The potential impact of measures to reduce GHG emissions from ships on exports: a statistical preliminary approach Report

[CR7] Centre for International Economy (2021) The potential impact of measures to reduce GHG emissions from ships on exports: a statistical preliminary approach Report

[CR8] Cepeda MA, Assis LF, Marujo LG, Caprace JD (2017). Effects of slow steaming strategies on a ship fleet. Marine Syst Ocean Technol.

[CR9] Cepeda MAFS, Caprace JD (2015) SIMULATING ECONOMICAL IMPACTS OF SLOW & ULTRA SLOW STEAMING STRATEGIES ON A BULK CARRIER FLEET. In: Proceeding of the XXIX Congresso de Pesquisa e Ensino em Transportes (pp 1052–1062).

[CR10] Dagkinis IOANNIS, Nikitakos NIKITAS (2015) Slow steaming options investigation using multi criteria decision analysis method. In: ECONSHIP 2015 Chios Greece.

[CR11] De Oliveira GF (2014). Determinants of European freight rates: the role of market power and trade imbalance. Transp Res Part E Logist Transp Rev.

[CR12] Doelle M, Chircop A (2019). Decarbonizing international shipping: an appraisal of the IMO's Initial Strategy. Rev Eur Comp Int Environ Law.

[CR13] Elzarka S, Morsi M (2014) The supply chain perspective on slow steaming. In: International Forum on Shipping, Ports and Airports (IFSPA) 2014: Sustainable Development in Shipping and Transport LogisticsHong Kong Polytechnic University.

[CR14] Faber J, Nelissen D, Hon G, Wang H, Tsimplis M (2012) Regulated slow steaming in maritime transport. An assessment of options, costs and benefits.

[CR15] Ferrari C, Parola F, Tei A (2015). Determinants of slow steaming and implications on service patterns. Marit Policy Manag.

[CR16] Finnsgård C, Kalantari J, Roso V, Woxenius J (2020). The Shipper's perspective on slow steaming-Study of Six Swedish companies. Transp Policy.

[CR17] Fourth IMO GHG Study (2020) Final Rep. IMO doc. MEPC 75/7/15.

[CR18] Gilani HA, Hoseinzadeh S (2021). Techno-economic study of compound parabolic collector in solar water heating system in the northern hemisphere. Appl Therm Eng.

[CR19] Gurning RS, Busse W, Lubnan M (2017) Decision Making of Full Speed, Slow Steaming, Extra Slow Steaming and Super Slow Steaming using TOPSIS. Int J Marine Eng Innov Res 2(1)

[CR20] Hämäläinen E (2014). Can slow steaming lower cost impacts of sulphur directive–shippers’ perspective. World Rev Intermodal Transp Res.

[CR21] Holland E, Nuttall P, Newell A, Prasad B, Veitayaki J, Bola A, Kaitu’u J (2014). Connecting the dots: policy connections between Pacific Island shipping and global CO2 and pollutant emission reduction. Carbon Manag.

[CR22] Hoseinzadeh S, Garcia DA (2022). Techno-economic assessment of hybrid energy flexibility systems for islands’ decarbonization: A case study in Italy. Sustain Energy Technol Assess.

[CR23] Hummels D, Schaur G (2012) Time as a trade barrier (No. w17758). National Bureau of Economic Research.

[CR24] Jiven K, Lammgård C, Woxenius J, Fridell E (2020) Förstudie initierad av Lighthouse, E. Consequences of speed reductions for ships.

[CR25] Karampampa IC (2014) THE IMPACT OF SLOW STEAMING ON SHIPPERS AND ON THEIR SUPPLY CHAINS: A WINDOW OF OPPORTUNITY FOR OTHER TRANSPORT MODES. Erasmus University Rotterdam.

[CR26] Khodayar Sahebi H, Hoseinzadeh S, Ghadamian H, Ghasemi MH, Esmaeilion F, Garcia DA (2021). Techno-economic analysis and new design of a photovoltaic power plant by a direct radiation amplification system. Sustainability.

[CR27] Kontovas CA, Psaraftis HN (2011). The link between economy and environment in the post-crisis era: lessons learned from slow steaming. Int J Decis Sci Risk Manag..

[CR28] Kontovas CA, Psaraftis HN (2011). The link between economy and environment in the post-crisis era: lessons learned from slow steaming. Int J Decis Sci Risk Manag.

[CR29] Lindstad H, Asbjørnslett BE, Strømman AH (2012). The Importance of economies of scale for reductions in greenhouse gas emissions from shipping. Energy Policy.

[CR30] Mallidis I, Iakovou E, Dekker R, Vlachos D (2018). The impact of slow steaming on the carriers’ and shippers’ costs: the case of a global logistics network. Transp Res Part E Logis Transp Rev.

[CR31] Maloni M, Paul JA, Gligor DM (2013). Slow steaming impacts on ocean carriers and shippers. Marit Econom Logist.

[CR32] Mander S (2017). Slow steaming and a new dawn for wind propulsion: a multi-level analysis of two low carbon shipping transitions. Mar Policy.

[CR33] Marine Environmental Protection Committee (MEPC) (2011) Reduction of GHG emissions from ships, Marginal abatement costs and cost effectiveness of energy efficiency measures. Submitted by (IMarEST). MEPC. 62/INF.7.

[CR34] Marine Environmental Protection Committee (MEPC) (2018) INITIAL IMO STRATEGY ON REDUCTION OF GHG EMISSIONS FROM SHIPS. MEPC 72/17. ADD.1

[CR35] Marine Environmental Protection Committee (MEPC) (2019) PROCEDURE FOR ASSESSING IMPACTS ON STATES OF CANDIDATE MEASURES. MEPC.1/Circ.885.

[CR36] Marine Environmental Protection Committee (MEPC) (2020) TERMS OF REFERENCE AND ARRANGEMENTS FOR THE CONDUCT OF A COMPREHENSIVE IMPACT ASSESSMENT OF THE SHORT-TERM MEASURE BEFORE MEPC 76. MEPC 75/18.

[CR37] Mimura N (2013). Sea-level rise caused by climate change and its implications for society. Proc Jpn Acad Ser B.

[CR38] Mundaca G, Strand J, Young IR (2021). Carbon pricing of international transport fuels: Impacts on carbon emissions and trade activity. J Environ Econ Manag.

[CR39] Newell A, Nuttall PM, Holland E (2015) Sustainable sea transport for the Pacific Islands: the obvious way forward. UN Sustainable Development Knowledge Platform

[CR40] Notteboom T, Pallis T, Rodrigue JP (2021). Disruptions and resilience in global container shipping and ports: the COVID-19 pandemic versus the 2008–2009 financial crisis. Marit Econ Logist.

[CR41] OECD. Making Development Co-operation Work for Small Island Developing States (2014) Retrieved from: Making Development Co-operation Work for Small Island Developing States | en | OECD

[CR42] Pradana MF, Hamdani MI, Noche B (2020). Shipping cost optimization on the Indonesian sea tollway due to weather. IOP Conf Ser Mater Sci Eng.

[CR43] Psaraftis HN, Zis T (2021) Impact assessment of a mandatory operational goal-based short-term measure to reduce GHG emissions from ships: the LDC/SIDS case study. In: International Environmental Agreements: Politics, Law and Economics (pp 1–23). 10.1007/s10784-020-09523-2 PMC7778843 33424522

[CR44] Psaraftis HN, Kontovas CA (2010). Balancing the economic and environmental performance of maritime transportation. Transp Res Part D Transp Environ.

[CR45] Psaraftis HN, Kontovas CA (2014). Ship speed optimization: concepts, models and combined speed-routing scenarios. Transp Res Part C Emerg Technol.

[CR46] Psaraftis HN, Kontovas CA (2020). Decarbonization of maritime transport: is there light at the end of the tunnel?. Sustainability.

[CR47] Robinson SA (2020). Climate change adaptation in SIDS: a systematic review of the literature pre and post the IPCC Fifth assessment report. Wiley Interdiscip Rev Clim Change.

[CR48] Sánchez RJ, Hoffmann J, Micco A, Pizzolitto GV, Sgut M, Wilmsmeier G (2003). Port efficiency and international trade: port efficiency as a determinant of maritime transport costs. Marit Econ Logist.

[CR49] Soyer B, Tettenborn A (2016) Slow steaming clauses and international sales contracts: a successful marriage?. In: International Trade and Carriage of Goods (pp 55–74). Informa Law from Routledge.

[CR50] UNCTAD (2014) Review of maritime transport. Chapter 6: Sustainable Freight Transport Development and Finance.

[CR51] UNCTAD (2021) Review of maritime transport. Retrieved from https://unctad.org/webflyer/review-maritime-transport-2021

[CR52] Vakili SV, Ölçer AI, Schönborn A (2021). Identification of shipyard priorities in a multi-criteria decision-making environment through a Transdisciplinary energy management framework: a real case study for a Turkish shipyard. J Marine Sci Eng.

[CR53] Vakili SV, Ballini F, Dalaklis D, Ölçer AI (2022). A Conceptual transdisciplinary framework to overcome energy efficiency barriers in ship operation cycles to meet imo’s initial green house gas strategy goals: case study for an iranian shipping company. Energies.

[CR54] Vakili S, Schönborn A, Ölçer AI (2022). Techno-economic feasibility of photovoltaic, wind and hybrid electrification systems for stand-alone and grid-connected shipyard electrification in Italy. J Clean Prod.

[CR55] Vakili S, Ölçer AI, Schönborn A, Ballini F, Hoang AT (2022c) Energy‐related clean and green framework for shipbuilding community towards zero‐emissions: a strategic analysis from concept to case study. Int J Energy Res.

[CR56] Valentine VF, Benamara H, Hoffmann J (2013). Maritime transport and international seaborne trade. Marit Policy Manag.

[CR57] Wiesmann A (2010). Slow steaming–a viable long-term option?. Wartsila Techn J.

[CR58] Wilmsmeier G, Sanchez RJ (2009). The relevance of international transport costs on food prices: endogenous and exogenous effects. Res Transp Econ.

[CR59] Wu WM (2020). The optimal speed in container shipping: theory and empirical evidence. Transp Res Part E Logist Transp Rev.

[CR60] Yin J, Fan L, Yang Z, Li KX (2014). Slow steaming of liner trade: its economic and environmental impacts. Marit Policy Manag.

[CR61] Zanne M, Počuča M, Bajec P (2013). Environmental and economic benefits of slow steaming. Trans Marit Sci.

[CR62] Zhao Y, Zhou J, Fan Y, Kuang H (2020) Sailing speed optimization model for slow steaming considering loss aversion mechanism. J Adv Transp.

